# Abstracts from Foreign Journals

**Published:** 1900-03

**Authors:** M. P. Ravenel

**Affiliations:** Philadelphia, Pa.


### ABSTRACTS FROM FOREIGN JOURNALS.

### FRENCH.

UNDER THE DIRECTION OF M. P. RAVENEL, M.D.
PHILADELPHIA, PA.

A PRODROMIC SYMPTOM OF RABIES IN THE BOVINE SPECIES. In a herd of cattle which had been attacked by a mad dog four young animals and one grown animal showed rabies after one month.

The following symptoms were noted : Irritation of the muzzle and upper lip, loss of appetite, cessation of rumination, constipation, a gloomy or even fierce aspect, abundant salivation, pawing, repeated bellowing, general tremors, paresis affecting especially the hind quarters, paralysis, and death.

The first of these symptoms—the irritation (twitching) of the upper lip—gives to the practitioner a prodromic symptom. It gives to the animal a grimace analogous to that of the rabbit, and continues during the whole course of the disease. It makes its appearance at least one day before loss of appetite is seen, and since it does not occur in any other affection it is of great value to the attending veterinarian.—*Recueil Vet.*, October, 1899.

THE ACTION OF CREOLIN IN ANTHRAX (by Hansen). The author was called to examine a cow taken suddenly sick. Respiration was difficult, fever quite high, and depression of vital forces very marked. Rumination had stopped, and secretion of milk was entirely arrested. The owner had already given some remedies to the animal, and had cut the tip of its ear. By its movements it had already sprinkled two cows alongside with blood. It died rapidly after having had one rectal hemorrhage. The post-mortem examination as well as the microscopical examination proved that the animal had died of anthrax.

Two days later a second cow became ill and showed the same symptoms as the first. Remembering the communication of Meier, the writer administered 30 grammes of creolin in a mucilaginous mixture. One hour after absorption of the medicine the animal showed a marked amelioration of its symptoms. The same evening a second dose of 20 grammes of creolin was given. On the next day the appetite had returned, the alarming symptoms disappeared, and at the end of seven days cure was complete. During this time the cow had been given a soupspoonful of creolin three time each day.

Some days after the second case had appeared the two cows which had been sprinkled with blood from the first animal showed alarming symptoms of the same nature as seen in the two preceding cases. The owner did not hesitate to administer creolin to them immediately, and also gave it as a prophylactic to all his other animals. The sick ones soon recovered, and no other case of anthrax occurred on the farm.—*Berliner Thieraerztl Wochenschrift*, 1899, No. 49; *Annales de Médecine Veterinaire*, March, 1900.

